# PAX6 does not regulate *Nfia* and *Nfib* expression during neocortical development

**DOI:** 10.1038/srep10668

**Published:** 2015-05-29

**Authors:** Jens Bunt, Jonathan W. C. Lim, Lu Zhao, Sharon Mason, Linda J. Richards

**Affiliations:** 1 Queensland Brain Institute, The University of Queensland, Brisbane, 4072, Australia; 2 The School of Biomedical Sciences, The University of Queensland, Brisbane, 4072, Australia

## Abstract

The Nuclear factor I (NFI) family of transcription factors regulates proliferation and differentiation throughout the developing central nervous system. In the developing telencephalon of humans and mice, reduced *Nfi* expression is associated with agenesis of the corpus callosum and other neurodevelopmental defects. Currently, little is known about how *Nfi* expression is regulated during early telencephalic development. PAX6, a transcription factor important for telencephalic development, has been proposed as an upstream regulator of *Nfi* expression in the neocortex. Here we demonstrate that, in the developing neocortex of mice, NFIA and NFIB are endogenously expressed in gradients with high caudo-medial to low rostro-lateral expression and are most highly expressed in the cortical plate. We found that this expression pattern deviates from that of PAX6, suggesting that PAX6 does not drive *Nfi* expression. This is supported by *in vitro* reporter assays showing that PAX6 over-expression does not regulate *Nfi* promoter activity. Similarly, we also found that in the *Pax6 Small Eye* mutant, no changes in *Nfi* mRNA or protein expression are observed in the neocortical ventricular zone where PAX6 and the NFIs are expressed. Together these data demonstrate that in mice, PAX6 is not a transcriptional activator of *Nfi* expression during neocortical development.

Transcriptional regulatory networks are fundamental to the development of distinct cellular populations throughout embryogenesis. These networks consist of interconnected and independent transcriptional regulatory cascades that are conventionally inferred from gene expression and phenotypic analyses of loss-of-function animal models. Identifying these regulatory pathways during neurodevelopment may implicate novel genes in neurodevelopmental disorders, or determine if similar disorders are the result of interconnected or distinct causes. In the developing telencephalon, the Nuclear factor I (NFI) family of transcription factors forms part of such an undefined network, with the majority of its regulatory components currently unknown. In mice the NFI transcription factors are expressed from embryonic day (E)12 within the dorsal telencephalon^1^, and function to regulate the proliferation and differentiation of neural progenitor cells^2^,^3^,^4^,^5^,^6^,^7^,^8^. Knockout mice for *Nfia*, *Nfib* or *Nfix* display similar forebrain phenotypes^2^,^3^,^4^,^5^,^6^,^7^,^8^. These mice are characterized by delayed glial differentiation, consequently resulting in malformations of the corpus callosum and hippocampus^2^,^3^,^4^,^5^,^6^,^7^,^8^. In humans, haploinsufficiency for *NFI* has been similarly associated with agenesis of the corpus callosum and other neurodevelopmental disorders^9^,^10^,^11^,^12^,^13^,^14^,^15^,^16^. Thus, precise regulation of *Nfi* expression is required for normal forebrain development in both humans and mice.

The transcription factor PAX6 has been implicated as a potential transcriptional activator of *Nfia* and *Nfib* expression in the developing neocortex^17^. PAX6 expression precedes both NFIA and NFIB, and is expressed within the proliferative ventricular zone (VZ) of the dorsal telencephalon throughout embryonic development^1^,^18^. The function of PAX6 has been examined in a number of different *Pax6* loss-of-function mouse models, most notably the *Pax6* *Small Eye* (*Pax6*^Sey^) mutant strain^19^,^20^,^21^. This model expresses a truncated PAX6 protein that lacks the homeobox DNA-binding domain due to a mutation that introduces a premature stop codon^20^. Gene expression array analyses comparing *Pax6*^Sey/Sey^ mutant and wildtype neocortices have identified potential downstream candidates regulated by PAX6 during prenatal development. In the *Pax6*^Sey/Sey^ neocortex, expression of both *Nfia* and *Nfib* are reduced at E12, with *Nfia* expression similarly reduced at E15^17^. As *Pax6* overexpression in cultured E13 cortical cells correspondingly increased *Nfia* expression, *Nfia* appears to be a *bona fide* transcriptional target of PAX6^17^. In this paper, we investigated the hypothesis that PAX6 functions as a transcriptional activator of *Nfia* and *Nfib* (hereafter referred to as *Nfi*) within the developing neocortex.

## Results

### NFI expression opposes the gradient of PAX6 expression during neocortical development

**Figure 1:**
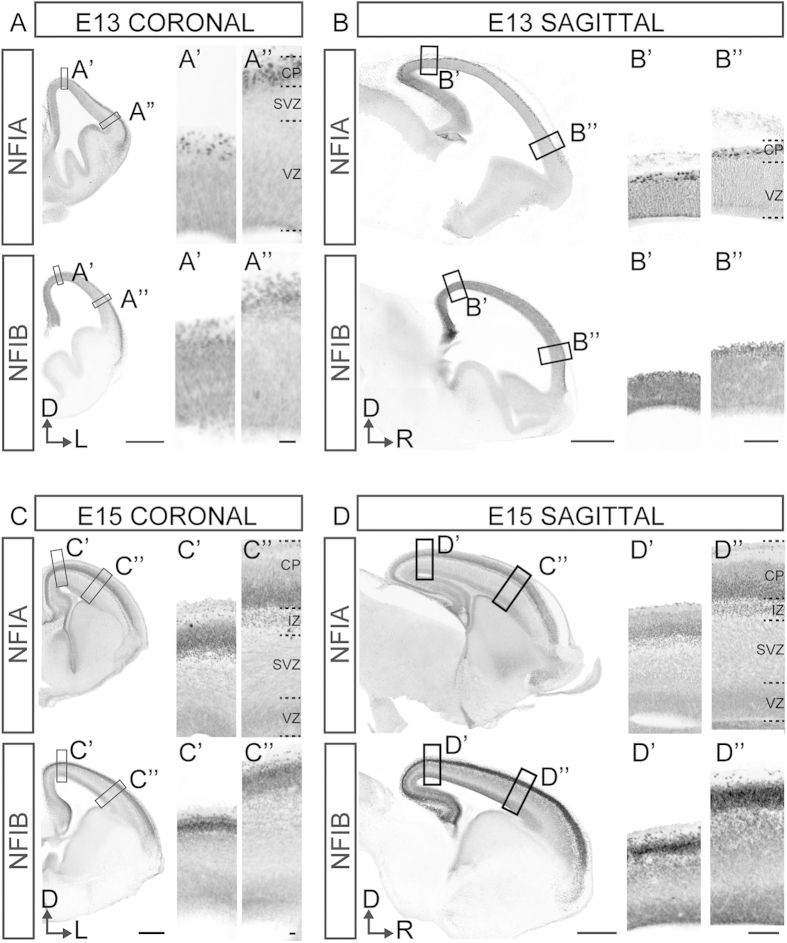
NFIA and NFIB are expressed in a high caudo-medial to low rostro-lateral gradient in the developing neocortex. Coronal and sagittal sections of E13 (**A** and **B**) and E15 (**C** and **D**) C57Bl/6J wildtype brains were analyzed for NFI protein expression in the medial-to-lateral and rostral-to-caudal axes respectively. NFIA and NFIB were expressed in a high medial to low lateral gradient at both ages (**A** and **C**) and in a high caudal to low rostral gradient at E13, but not at E15 (**B** and **D**). The developing cortical plate (CP) showed highest expression across the cortical laminae, with intermediate expression in the ventricular zone (VZ). For coronal sections, **A**’ and **C**’ represent medial insets and **A**” and **C**” represent lateral insets. For sagittal sections, **B**’ and **D**’ represent caudal insets and **B**” and **D**” represent rostral insets. Scale bars represent 500 μm in low-powered magnifications and 100 μm (**A** and **C**) or 20 μm (**B** and **D**) in high-powered magnification panels. SVZ = subventricular zone; IZ = intermediate zone; D = dorsal; L = lateral; R = rostral.

It has previously been shown that PAX6 expression in the developing neocortex is limited to the VZ, where it is expressed in a high rostro-lateral to low caudo-medial gradient^22^. We reasoned that if PAX6 transcriptionally activates *Nfia* or *Nfib* expression, the expression patterns of these transcription factors would resemble each other. To test this, we analyzed NFI protein expression across the neocortex of wildtype C57Bl/6J mice at E13 and E15. In contrast to PAX6 expression, we observed that in coronal sections at E13, both NFIA and NFIB are expressed in high medial to low lateral gradients (Fig. 1A). Similarly, analyses of sagittal sections at this age show that both NFIA and NFIB are expressed higher caudally and lower rostrally (Fig. 1B). At E15, the graded expression of NFIA and NFIB is sustained in the medial-to-lateral axis (Fig. 1C), but not in the rostral-to-caudal axis, where NFI protein expression was now more evenly distributed (Fig. 1D). The graded expression of *Nfia* and *Nfib* in the sagittal plane was confirmed at the mRNA level at E11.5 and E13.5 by examining *in situ* hybridization data available from the Allen Brain Atlas (Supplementary Figure 1)^23^.

Similarly, we note that the NFI transcription factors were more broadly expressed across the cortical laminae as compared to PAX6 expression. In line with our previous characterization of NFI expression in the developing forebrain^1^, we observed that both NFIA and NFIB are most highly expressed within the cortical plate of the neocortex (Fig. 1 and S1), where PAX6 is not expressed^18^. Thus, in mice, the graded expression pattern of NFIA and NFIB across the wildtype neocortex does not resemble that of PAX6 expression.

**Figure 2:**
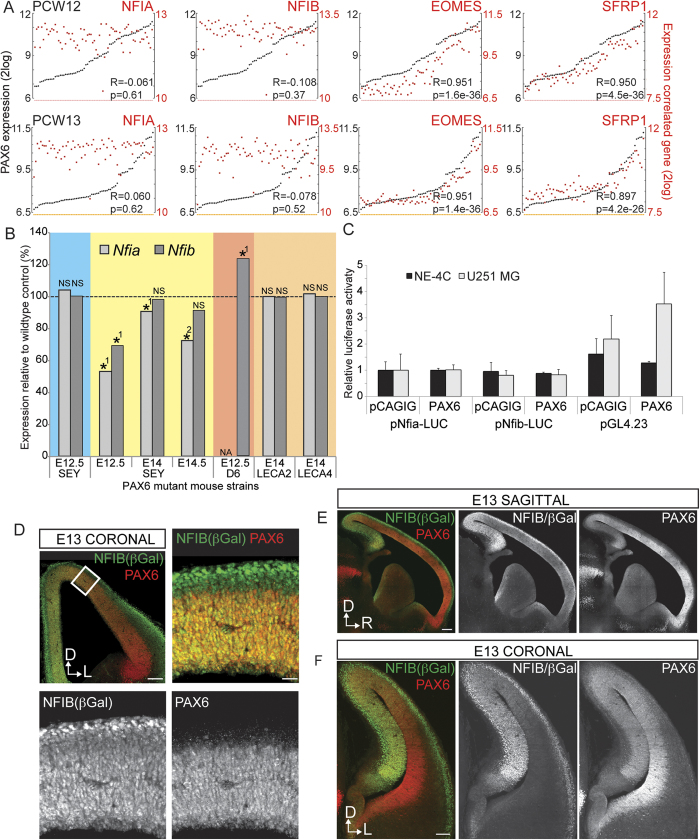
NFI and PAX6 expression are not correlated in the developing neocortex and PAX6 does not activate *Nfi* promoter activity *in vitro*. (**A**). Correlation of *PAX6* mRNA expression (black) with *NFIA, NFIB, EOMES* or *SFRP1* mRNA expression (red) at 12 (upper panels) and 13 (lower panels) weeks post-conception in the human neocortex^24^. (**B**). Relative expression of *Nfia* and *Nfib* in the *Pax6* loss-of-function mouse models *Pax6*^Sey^ (SEY), *Pax6*^Leca2^ (LECA2) and *Pax6*^Leca4^ (LECA4) and the transgenic PAX6 over-expression model Dach1-*PAX6* (D6-PAX6) from published expression datasets^27^,^29^,^30^,^34^, normalized to wildtype control (100%; dashed line) for comparison between datasets. All datasets represent rostral cortex/neocortex, with the exception of the first set (blue) that was generated from the PAX6-positive fraction only^34^. Statistical significance was determined using one-way ANOVA for genotypes with n = 3 or more. NA = raw data not available from original dataset, *^1^ = reported significant in original dataset, *^2^ = p < 0.05 by one-way ANOVA, NS = non-significant (one-way ANOVA). (**C**). Relative luciferase activity of NE-4C and U251 MG cells transfected with the pGL4.23 luciferase construct driven by the mouse *Nfia* or *Nfib* promoter (pNfia-LUC and pNfib-LUC respectively) or its endogenous minimal promoter. PAX6 and pCAGIG denote cells co-transfected with the PAX6-over-expression or empty control vectors. Luciferase activity was determined 48 hours after transfection and normalized to *Renilla* luciferase that was co-trasfected as an internal control. pNfia-LUC with pCAGIG was scaled to ‘1’ and all other conditions were then normalized to this to determine relative luciferase activity. No statistical significance was observed between pCAGIG and PAX6 for all experimental conditions (n = 3, figure depicts a representative sample with three technical replicates). (**D-F**). Representative immunofluorescence images of coronal (**D** and **F**) and sagittal (**E**) sections of E13 *Nfib* heterozygous brains stained for PAX6 and the β-galactoside reporter knocked into the *Nfib* locus. The boxed region in the low-magnification panel in (**D**) represents subsequent panels with high-powered magnification showing PAX6 and β-galactoside co-expression. Scale bars represent 100 μm (low-powered) and 20 μm (high-powered magnification) in D, 200 μm (**E**) or 100 μm (**F**). D = dorsal; R = rostral; L = lateral.

To determine whether *NFI* expression correlates with *PAX6* expression during human neocortical development, we analyzed publically available mRNA expression data obtained from neocortical regions at 12 and 13 weeks post-conception^24^ (Fig. 2A), which correspond to our E13 and E15 analyses in mice^25^. At both ages, *NFIA* and *NFIB* expression levels in the neocortical areas showed no significant correlation with *PAX6* expression levels in the neocortex (Fig. 2A), whereas *SFRP1* and *EOMES* showed significant correlation as reported previously in mice^17^,^26^,^27^. Thus, endogenous expression of *NFIA* and *NFIB* do not appear to be positively correlated with PAX6 expression in the developing neocortex of both mice and humans.

### Differential expression of *Nfia* and *Nfib* in *Pax6* mouse models

We next investigated the reproducibility of reduced *Nfi* expression previously reported in microarray analyses of the *Pax6*^Sey/Sey^ mutant^17^. Using validated independent primer sets for quantitative PCR^28^, we analyzed *Nfi* mRNA levels in microdissected dorsolateral cortex/neocortex of E13 *Pax6*^Sey/Sey^ and wildtype embryos. We observed reduced *Nfia* and *Nfib* expression in the *Pax6*^Sey/Sey^ neocortex (-3.2 and -4.3 fold, respectively when compared within litters). These reductions did not achieve statistical significance when litters were pooled due to the large variation between litters (p = 0.32 and p = 0.22, respectively; one-way ANOVA). To determine if the down-regulation of *Nfi* expression observed in the *Pax6*^Sey/Sey^ neocortex is reproducible in microarray analyses, we analyzed independent *Pax6*^Sey/Sey^ expression datasets of E12.5 neocortex^27^, E14 rostral cortex^29^ and E14.5 neocortex^30^ (Fig. 2B; yellow). In line with the observations of Holm *et al.*^17^, *Nfia* expression was significantly reduced at all ages. In contrast, *Nfib* expression was significantly reduced only in the E12.5 dataset.

As the reduction of *Nfi* expression may be specific to the *Pax6*^Sey^ mouse model and its phenotype, we also analyzed available expression data from other *Pax6* mutants, namely the D6-PAX6^27^, *Pax6*^Leca2^ and *Pax6*^Leca4^ 31,32 mouse mutants. A significant increase in *Nfib* expression was observed in E12.5 neocortex for the D6-PAX6 mouse model that over-expresses wildtype PAX6 (Fig. 2B; red, Sansom and colleagues also reported increased *Nfia* expression but no raw data is available to validate this)^27^. In the *Pax6*^Leca2^ and *Pax6*^Leca4^ mouse models, no reduction in *Nfia* or *Nfib* expression was detected in E14 rostral cortex in contrast to reduced *Nfia* expression in the *Pax6*^Sey^ rostral cortex collected and analyzed within the same experiment (Fig. 2B; orange)^29^. These mice differ from the *Pax6*^Sey^ mice as they both carry single missense mutations that result in the expression of functional PAX6 protein with reduced DNA binding capability^31^,^32^. Thus the mis-regulation of *Nfi* expression is not consistently observed in other *Pax6* mouse models.

### Mutation of PAX6 does not alter radial glial expression of NFI

Our analyses thus far suggest that PAX6 is unlikely to function as a direct transcriptional activator of *Nfi* expression in the developing neocortex. To further test this hypothesis, we used an *in vitro* reporter assay to determine if PAX6 over-expression regulates *Nfi* promoter activity. We transfected mouse *Nfia* or *Nfib* promoter-driven luciferase constructs (pNfia-LUC or pNfib-LUC), or a control reporter plasmid into the NE-4C (mouse neuroepithelial cell line) and U251 MG (human glioma cell line) cell lines. No change in *Nfi* promoter activities were observed when these cells were co-transfected with a PAX6 overexpression construct (pCAGIG-PAX6) as compared to an empty control vector (Fig. 2C).

Nevertheless, if PAX6 autonomously regulates *Nfi* expression *in vivo*, this regulation would likely be limited to the VZ where PAX6 and the NFIs are expressed^22^. To test this, we first determined whether PAX6 and NFIB are co-expressed within the same cells in the neocortical VZ. Using a knock-in reporter gene for NFIB substituted into the deleted exon of the *Nfib* knockout mice^3^,^7^,^33^, we observed co-expression of the β-galactosidase reporter gene and PAX6 in the radial glial cells of E13 heterozygous *Nfib* knockout mice, as has been reported at E18.5 (Fig. 2D)^3^,^7^,^33^. In keeping with our analyses of endogenous NFI expression in wildtype mice (Fig. 1), PAX6 expression across the neocortical VZ negatively correlated with β-galactosidase expression in *Nfib* heterozygous mice when observed in both the sagittal (Fig. 2E) and coronal (Fig. 2F) planes.

To investigate whether *Nfi* expression is specifically mis-regulated in the radial glial cells of *Pax6*^Sey^ mice, we analyzed *Nfi* expression using previously published data obtained from flow cytometry-sorted populations of PAX6-positive cells. Price and colleagues generated this data by crossing the *Pax6*^Sey^ mice to the DTy54 reporter mouse line with GFP expression in PAX6-positive cells^34^. Analyses of *Nfia* and *Nfib* expression in GFP-positive cells obtained from E12.5 *Pax6*^Sey/Sey^ mutant and wildtype neocortices revealed no difference in *Nfi* expression (Fig. 2B; blue). Thus, the reduction of *Nfi* expression observed in microarray analyses of *Pax6*^Sey/Sey^ mutants cannot be justified by the mis-regulation of *Nfi* expression in radial glial cells.

**Figure 3:**
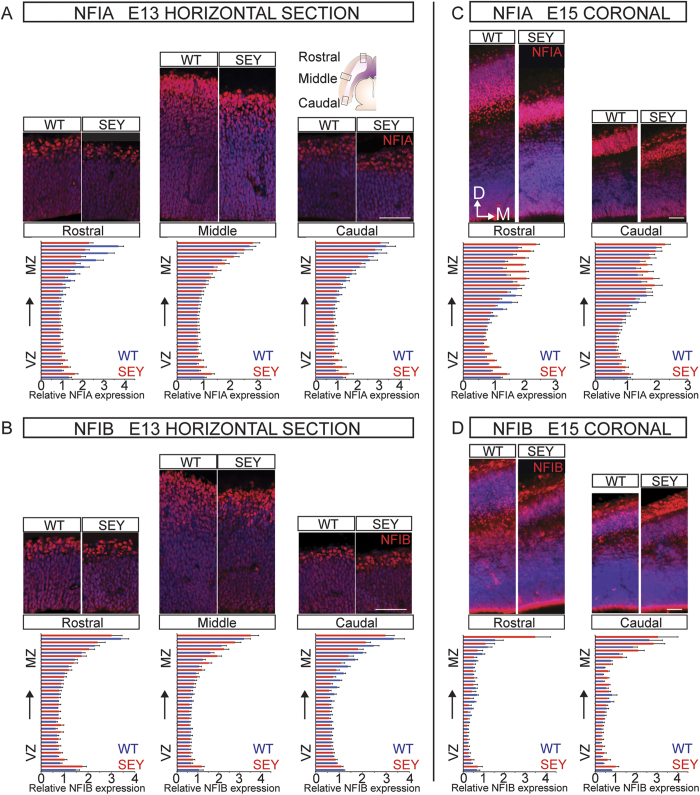
NFI protein expression is not reduced in the ventricular zone of E13 and E15 *Pax6*^Sey/Sey^ neocortex. Representative immunofluorescence images of rostral, middle and caudal cortical regions stained for NFIA (A; red) or NFIB (B; red) and co-stained for DAPI (blue) in E13 wildtype (WT) and *Pax6*^Sey/Sey^ mutant (SEY) horizontal brain sections representing the middle of the dorsal telencephalon. Fluorescence intensity histograms represent the mean fluorescence intensity of NFIA (**A**) or NFIB (**B**) expression, divided into 20 equally spaced bins from the ventricular to pial surfaces and normalized to fluorescence intensity of DAPI within each bin (n = 6 per genotype). Mean fluorescence intensity for *Pax6*^Sey/Sey^ is represented in red with wildtype in blue. NFIA and NFIB showed no significant change in expression in any of the bins that represented the ventricular zone of *Pax6*^Sey/Sey^ mutants. (**C D**). Similar analyses were performed using E15 *Pax6*^Sey/Sey^ and wildtype brains sectioned coronally, with matched rostral and caudal sections analyzed independently. Similar to E13, NFIA (**C**) and NFIB (**D**) expression were not significantly changed in bins corresponding to the ventricular zone, but NFI expression was higher in the cortical plate of the *Pax6*^Sey/Sey^ mutant. Error bars represent standard error of the mean. VZ = ventricular zone, MZ = marginal zone, D = dorsal, M = medial. Scale bar represents 50 μm.

To validate this at the protein level, we quantified NFIA and NFIB protein expression in horizontal sections of E13 *Pax6*^Sey/Sey^ mutant and wildtype brains (Fig. 3A,B). For each section analyzed, the neocortex from the ventricular to pial surfaces were divided into 20 equally spaced bins, with strict precautions taken to account for variability between sections (see Methods for a full description). These analyses were independently repeated at three different regions along the rostral to caudal axis to account for the graded expression observed in this axis. In all three analyses, we observed no significant reduction in NFI expression in the bins that represent the VZ where PAX6 is expressed. On the contrary, slight changes in NFIA expression were observed in the bins that corresponded to the cortical plate where PAX6 is not expressed but the NFI transcription factors are strongly expressed.

We conducted similar analyses on E15 *Pax6*^Sey/Sey^ mutant brains sectioned coronally, and independently analyzed matched rostral and caudal sections. Again, no significant decrease in NFI expression was detected in bins that correspond to the VZ (Fig. 3C,D). In the bins that represented the cortical plate, we observed that peak NFIA expression shifted dorsally towards the pial surface in the *Pax6*^Sey/Sey^ mutants as compared to wildtype controls. Nevertheless, as the VZ cells that express PAX6 and the NFI transcription factors showed no mis-regulation of NFI expression, PAX6 does not function to activate *Nfi* transcription in radial glial cells of the developing neocortex.

## Discussion

The identification of transcriptional regulatory cascades is integral to determining whether similar neurodevelopmental disorders are the result of interconnected or distinct causes. In the developing telencephalon, the NFI and PAX6 transcription factors are involved in proliferation and neurogenesis^2^,^3^,^4^,^5^,^6^,^7^,^8^,^27^,^29^,^34^,^35^,^36^,^37^. Haploinsufficiency of these transcription factors in humans is similarly implicated with agenesis of the corpus callosum^9^,^10^,^11^,^12^,^13^,^14^,^15^,^16^,^38^,^39^. As PAX6 expression precedes NFI expression in the neocortex^1^,^18^ and deletion of *Pax6* results in reduced *Nfi* expression in the *Pax6*^Sey/Sey^ neocortex^17^, PAX6 was hypothesized to directly regulate *Nfi* expression during neocortical development.

In the current study, we present several lines of evidence that contradict this hypothesis. Whilst gene expression analyses of *Pax6*^Sey/Sey^ neocortex validate the reduction of *Nfi* expression reported previously^17^,^27^,^29^,^30^, our immunohistochemistry analyses to investigate NFI expression, and the inconsistencies in *Nfi* expression observed amongst the *Pax6* mouse models suggest possible discrepancies^27^,^32^. Significantly, we report from our analyses as well as the data published by Price and colleagues^34^ that *Nfia* and *Nfib* are not mis-regulated at the mRNA or protein levels in the *Pax6*^Sey/Sey^ VZ where PAX6 may autonomously regulate these transcription factors. This suggests that, despite the reduction of *Nfi* expression observed in the *Pax6*^Sey/Sey^ neocortex, PAX6 is not a direct transcriptional activator of *Nfi* expression in the developing neocortex.

Interestingly, we observed increased NFIA expression in the cortical plate of *Pax6*^Sey/Sey^ mutants (Fig. 3C). As PAX6 is not expressed in the developing cortical plate, PAX6 is unlikely to transcriptionally activate *Nfia* expression in this region. Rather, it is likely that the previously described precocious neurogenesis in the *Pax6*^Sey/Sey^ mutant results in a thinner cortical plate that is disproportionately populated by deep layer neurons^35^,^37^,^40^,^41^,^42^. As NFIA is more highly expressed in the deep layer neurons as compared to upper layer neurons^1^,^3^, this could account for the increased NFI protein expression observed within this region.

Our findings in this study are supported by previous independent chromatin immunoprecipitation experiments suggesting that *Nfia* and *Nfib* are not direct targets of PAX6 during neocortical development. Chromatin immunoprecipitation for PAX6 using E12.5 neocortical and E15.5 forebrain tissues does not detect binding of this transcription factor to the *Nfi* promoter regions^27^,^43^. Interestingly, binding of PAX6 at the *Nfia* and *Nfib* promoter regions in mice was detected for the pancreatic βT3 cells^43^ and the adult olfactory bulb^44^, respectively.

Similarly, our study is also supported by the phenotypes observed upon deletion of *Nfi* in mice, or *NFI* haploinsufficiency in humans as compared to that observed with PAX6 deficiency. For instance, PAX6 deficiency in humans and mice is associated with microcephaly^45^,^46^,^47^. In contrast, NFI reduction is implicated in ventriculomegaly and macrocephaly^9^,^10^,^11^,^12^. These opposite phenotypes suggest that if PAX6 were to regulate *Nfi* expression, it would likely function as a transcriptional repressor. However, no increase in *Nfi* expression was observed in the VZ of the *Pax6*^Sey/Sey^ neocortex by mRNA expression (Fig. 2B) or immunohistochemistry analyses (Fig. 3). Taken together, this evidence indicates that PAX6 is not a direct regulator of *Nfi* expression during neocortical development.

While our study demonstrates that down-regulation of expression does not necessarily imply a direct transcriptional relationship, similar precautions are required to prevent over-interpretation of our findings. Specifically, binding of PAX6 to the *Nfi* promoter in pancreatic βT3 cells^43^ and the adult olfactory bulb^44^ suggest that PAX6 may regulate *Nfi* expression within other tissue types or perhaps even within the neocortex at ages not examined. Interestingly, we note that within our analyses of E13 *Nfib* heterozygous mice, high expression of both PAX6 and β-galactosidase was present within the hippocampal primordia (Fig. 2E,F). However, as our analyses were strictly restricted to the developing neocortex, whether PAX6 regulates *Nfi* transcription during hippocampal development necessitates further investigation.

Although we show that PAX6 is not a direct transcriptional activator of *Nfi* expression in the neocortex, an interesting area of future study would be to determine the mechanism underlying the reduced *Nfi* expression observed in the *Pax6*^Sey/Sey^ neocortex. We propose an alternative hypothesis for this based on previous studies of the *Pax6*^Sey/Sey^ mutant. As PAX6 is involved in establishing the boundary between the dorsal and ventral telencephalon^22^,^48^,^49^,^50^,^51^, and is required for normal cell cycle exit and cortical cell identity specification^35^,^37^,^40^, the reduced *Nfi* expression observed in the *Pax6*^Sey/Sey^ mutant may represent a change in the cellular composition of the tissue analyzed in gene expression analyses. For example, *Nfi* expression may be indirectly reduced as radial glial cells that lacked functional PAX6 expression may differentiate prematurely or transition towards a ventral telencephalic progenitor cell fate^22^,^35^,^37^,^40^,^48^,^49^,^50^,^51^. This in turn results in the thinner cortical plate observed in *Pax6*^Sey/Sey^ mutants^35^,^37^,^40^,^41^,^42^. As NFIA and NFIB are expressed in the VZ and the cortical plate of the developing neocortex, these changes in cellular composition may account for the reduced expression of these transcription factors.

To conclude, by analyzing NFI expression in the wildtype and *Pax6*^Sey/Sey^ neocortices, we determined that PAX6 does not directly regulate *Nfi* expression during neocortical development. Our findings demonstrate the importance of careful phenotypic analyses when interpreting transcriptional regulatory cascades predicted from mixed cellular populations.

## Methods

### Animal breeding and tissue collection

All mice used in this study were bred at The University of Queensland with approval from the institutional Animal Ethics Committee and all experiments were performed in accordance with the approved guidelines. *Nfib* knockout mice^33^ and *Pax6*^Sey-neu^ (referred to as *Pax6*^Sey^)^19^ (original breeding stocks were a kind gift from Professor Grant Mastick, University of Nevada, Reno) were maintained on the C57Bl/6J and 102/El x C3H/Rl x C57Bl/6J backgrounds respectively. To generate timed-pregnant females, male and female mice were placed together overnight and checked the following day for vaginal plugs. This day was designated as E0 if a vaginal plug was present. Embryos were removed from dams that were placed under anesthesia using sodium pentobarbital (Abbott Laboratories), and were either drop fixed (E13 embryos) or transcardially perfused (E15 embryos) with 0.9% (w/w) saline, followed by 4% (w/w) paraformaldehyde in 1x phosphate-buffered saline (PBS; pH 7.4) for further analyses. For mRNA expression analyses of E13 neocortical tissue, the dorsolateral cortex (excluding the medial cortex) was microdissected in ice-cold sterile PBS and immediately snap frozen for mRNA isolation.

### Immunohistochemistry

Brains of paraformaldehyde-fixed embryos were removed from the skull and sectioned coronally, sagittally or horizontally at 50 μm using a vibratome. Chromogenic immunohistochemistry (IHC) or fluorescence immunohistochemistry (IF) was conducted as previously described with minor modifications^1^. Primary antibodies used for immunohistochemistry were as follows: rabbit anti-NFIA (1:500; ARP32714, Aviva Systems Biology (IF) or 1:10,000; HPA008884, Sigma (IHC)), rabbit anti-NFIB (1:500; ARP32716, Aviva Systems Biology or 1:10,000; HPA003956, Sigma), chicken anti-β-galactosidase (1:500; ab9361, Abcam (IF)) and rabbit anti-PAX6 (1:500; ab2237, Abcam (IF)). For IHC, a biotinylated goat anti-rabbit secondary antibody (1:500; BA-5000, Vector Laboratories) was used, followed by incubation with the VECTASTAIN elite ABC kit (Vector Laboratories) and nickel DAB staining as previously described^1^. For IF, Alexa Fluor 488-conjugated goat anti-chicken (1:500; A-11039, Invitrogen) and Alexa Fluor 555-conjugated goat anti-rabbit (1:500; A-11034, Invitrogen) secondary antibodies were used for detection. Immunofluorescent sections were counterstained with 4`,6-diamidino-2-phenylindole, dihydrochloride (DAPI, Invitrogen) to label cell nuclei and coverslipped using ProLong Gold anti-fade reagent (Invitrogen). To minimize variability for IF quantification, matched sections that were to be compared between *Pax6*^Sey/Sey^ and wildtype littermates were mounted on the same slide to ensure identical staining conditions.

### Imaging and data analysis

Brightfield and wide-field fluorescence imaging was performed with a Zeiss upright Axio-Imager Z1 microscope fitted with Axio- Cam HRc and HRm cameras and Apotome. Images were acquired with AxioVision software (Carl Zeiss) or ZEN blue (Carl Zeiss). For IF, matched sections from 6 brains were imaged per condition using identical acquisition settings to allow for quantification. Following this, matched neocortical regions of 100 μm width were cropped from each section in Photoshop (Adobe) and imported into ImageJ software (NIH). To generate fluorescence intensity histograms, cropped sections were divided into 20 bins of equal size across the ventricular to pial surfaces and fluorescence intensity corresponding to NFIA or NFIB was normalized to DAPI intensity within each bin. Statistical significance was determined using one-way ANOVA, with *p*-values below 0.05 considered significant. All values are presented as the mean, with error bars representing the standard error of the mean. Images in figures are representative images that have been identically cropped, enhanced for contrast and brightness, and pseudo-colored to permit overlay using Adobe Photoshop software.

### Quantification of *Nfi* mRNA expression

Quantitative PCR was performed as previously described^52^. Briefly, RNA of snap-frozen E13 neocortex of *Pax6*^Sey^ mutant or wildtype littermates of 3 litters was isolated using TRIzol reagent (Invitrogen) and 1 μg of RNA was reverse transcribed using SuperScript III First-Strand Synthesis SuperMix (Invitrogen) and Oligo(dT) primers (Invitrogen) as per the manufacturer’s instructions. Diluted samples were used for real-time qPCR with Platinum SYBR Green qPCR SuperMix (Invitrogen) and 0.25 μM forward and reverse primers. The qPCR reaction was carried out on a Rotor-Gene 3000 (Corbett Life Science) with the following thermocycler conditions: 10 minutes at 95 °C followed by 40 cycles with 15 seconds denaturation at 95 °C, 20 seconds annealing at 60 °C, and 30 seconds extension at 72 °C. Relative expression was determined using the ΔΔCt method with the housekeeping gene beta-2-microglobulin used as a relative standard. Statistical analyses were performed using one-way ANOVA. Error bars represent the standard error of the mean. Primers were as previously described^28^: B2MM 5`-agactgatacatacgcctgcag-3`, B2MMC 5`-gcaggttcaaatgaatcttcag-3`, mNFI-AE23 5`-ggcatactttgtacatgcagc-3`, mNFI-AE4C2 5`-acctgatgtgacaaagctgtcc-3`, mNFI-BE2 5`-gtttttggcatactacgtgcagg-3` and mNFI-BE3C 5`-ctctgatacattgaagactccg-3`.

### Dual-luciferase reporter assays

The U251 MG human glioblastoma^53^ and NE-4C mouse neuroepithelial^54^ cell lines were obtained from American Type Culture Collection (ATCC) and cultured in DMEM medium (Invitrogen), supplemented with 10% fetal bovine serum, 100 U/mL penicillin and 100 μg/mL streptomycin (Invitrogen) at 37 °C in a humidified atmosphere containing 5% CO_2_. The mouse *Nfia* (2,329 base pairs) and *Nfib* (2,484 base pairs) promoters were cloned into the pGL4.23 luciferase construct (Promega) to drive firefly luciferase expression, using primers with added NheI (forward primer) and BglII (reverse primer) restrictions sites; pNfia_F 5`-ttacatgttggttccctcagc-3`, pNfia_R 5`-tgcttacctgggtgagacag-3` pNfib_F 5`-ccaggctcttggtttacagg-3`, pNfib_R 5`-tcgccttaaaacgcactttc-3’. The mouse PAX6 coding sequence was cloned into pCAGIG (Addgene) from pMXIG-Pax6^55^, kindly provided by Professor Magdalena Gotz. Cells were seeded into a 96-well plate 24 hours prior to transfection at a density of 3,000 cells per well. pNfia-Luc, pNfib-Luc or the control pGL4.23 plasmid were co-transfected with either pCAGIG-PAX6 or pCAGIG into seeded cells using FuGENE HD (Promega). Renilla luciferase (pRL-SV40, Promega) was co-transfected with all transfections as an internal control for normalization of firefly luciferase activity. Luciferase activity was assayed 48 hours after transfection using the Dual-Luciferase Reporter Assay System (Promega). All experimental conditions were performed as three independent experiments of triplicates, of which one is represented. Statistical significance was determined using a one-way ANOVA.

### Microarray expression data analysis

Expression data of *Pax6*^Sey^ mutant E12.5 neocortical radial glial cells (GSE38703)^34^, *Pax6*^Sey^, *Pax6*^Leca2^ and *Pax6*^Leca4^ mutant E14 rostral cortex (GSE35260)^29^ and *Pax6*^Sey^ mutant E14.5 neocortex (GSE32271)^30^ were obtained from the Gene Expression Omnibus database. The processed data in ^2^log scale were converted to absolute scale and the difference in expression of *Nfia* or *Nfib* as compared to the wildtype embryos was calculated and represented as a percentage of the expression in wildtype controls. When more than 3 samples were available, statistical significance was determined using one-way ANOVA. Expression in human cortex at 12 and 13 weeks post-conception was obtained from the GSE25219 dataset^24^ and analyzed and visualized using the R2: microarray analysis and visualization platform ( http://r2.amc.nl) as previously described^56^.

## Additional Information

**How to cite this article**: Bunt, J. *et al.* PAX6 does not regulate *Nfia* and *Nfib* expression during neocortical development. *Sci. Rep.* **5**, 10668; doi: 10.1038/srep10668 (2015).

## Supplementary Material

Supplementary Information
