# Supplementary material for: PAX6 does not regulate Nfia and Nfib expression during neocortical development
Source: Sci Rep. 2015 May 29;5:10668. doi: 10.1038/srep10668 (PMC4448127; doi:10.1038/srep10668)
Supplement: Supplementary Information [file srep10668-s1.pdf]

Supplementary information

PAX6 does not regulate *Nfia* and *Nfib* expression during neocortical development

Jens Bunt[1], Jonathan W. C. Lim[1], Lu Zhao[1], Sharon Mason[1] and Linda J. Richards[1,2*]
[1]Queensland Brain Institute, and [2]The School of Biomedical Sciences, The University of Queensland, Brisbane, 4072, Australia.

*Author for correspondence:
Professor Linda J. Richards, PhD
NHMRC Principal Research Fellow
Head, Cortical Development and Axon Guidance Laboratory,
The Queensland Brain Institute and The School of Biomedical Sciences
The University of Queensland, St Lucia, 4072, AUSTRALIA
ph: +61 7 33466355
fax: +61 7 33466301
email: richards@uq.edu.au

Supplementary Figure S1
Bunt *et al*

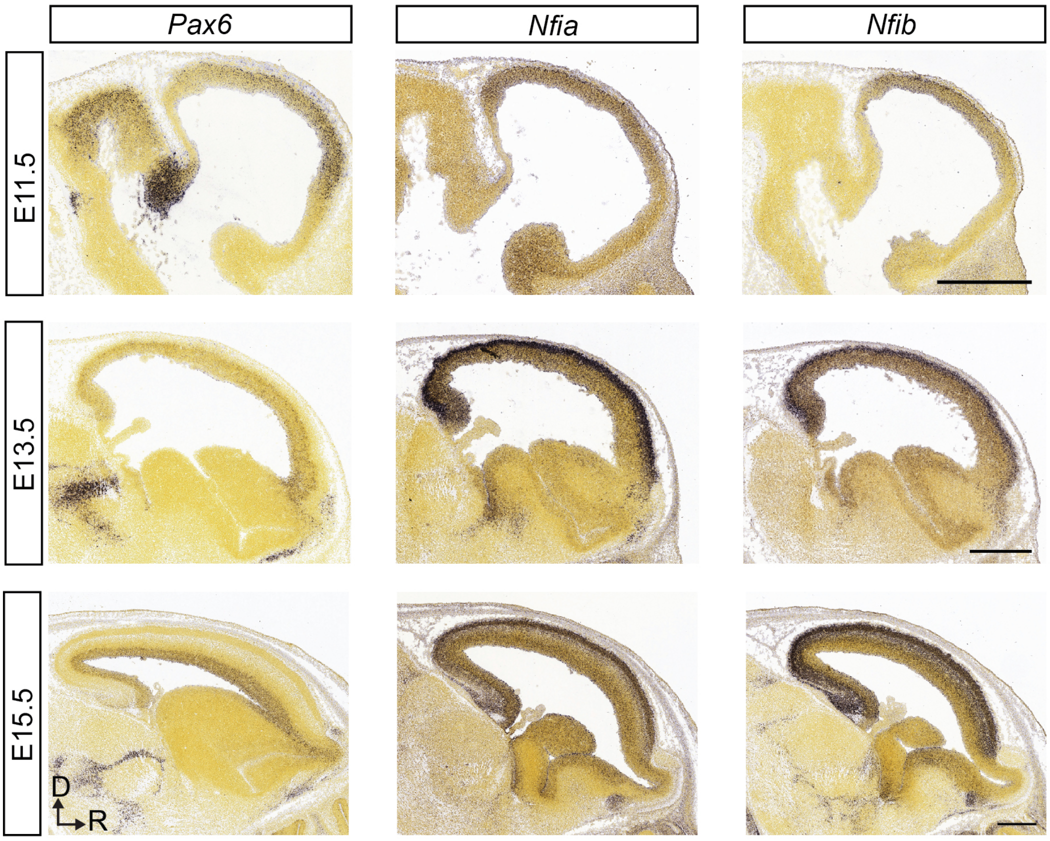

Supplementary Figure S1. ***Nfia*** **and** ***Nfib*** **mRNA are expressed in a high caudal to low rostral gradient during early neocortical development.** *In situ* hybridization analyses using probes for *Pax6*, *Nfia* and *Nfib* were obtained from the Allen Brain Atlas[23]. Analyses were performed using sagittal sections of E11.5, E13.5 and E15.5 C57Bl/6J wildtype brains. mRNA expression of all analyzed genes were detected from E11.5. While *Pax6* was expressed in a high rostral to low caudal gradient in the ventricular zone of the neocortex at all ages, *Nfia* and *Nfib* were expressed in opposing high caudal to low rostral gradients at E11.5 and E13.5, with highest expression observed in the developing cortical plate. At E15.5, the expression of *Nfia* and *Nfib* remains the highest in the cortical plate, but expression across the rostral-to-caudal axis is now more evenly distributed. Scale bar represents 500 μm. D = dorsal; R = rostral. Image credit: Allen Institute for Brain Science.
